# Socio-cultural factors, gender roles and religious ideologies contributing to Caesarian-section refusal in Nigeria

**DOI:** 10.1186/s12978-015-0050-7

**Published:** 2015-08-12

**Authors:** Nnanna U. Ugwu, Bregje de Kok

**Affiliations:** Health and development consultant Catholic Centre for Life/St Joseph’s Catholic hospital, P. O. Box 28, Ijebu-Igbo, Ogun State Nigeria; Lecturer & ISRF Research Fellow Institute for International Health and Development, Queen Margaret University, Queen Margaret University Drive, EH21 6UU Musselburgh, United Kingdom

**Keywords:** Maternal health, Maternal mortality, Childbirth, Caesarian section, Socio-cultural meanings, Gender, Religion, Alternative providers, Nigeria

## Abstract

**Background:**

The death of women from pregnancy-related causes is a serious challenge that international development initiatives, including the Millennium Development Goals, have been trying to redress for decades. The majority of these pregnancy-related deaths occur in developing countries especially in Sub-Saharan Africa. The provision of Emergency Obstetric Care (EmOC), including Caesarean section (CS) has been identified as one of the key ingredients necessary for the reduction of high maternal mortality ratios. However, it appears that creating access to EmOC facilities is not all that is required to reduce maternal mortality: socio-cultural issues in Sub-Saharan countries including Nigeria seem to deter women from accepting CS. This study seeks to explore some of the socio-cultural concerns that reinforce delays and non-acceptance of CS in a Nigerian community.

**Methods:**

This is a mixed method study that combined both qualitative and quantitative strategies of enquiry. The hospital’s delivery records from 2006–2010 provided data for quantitative analysis. This quantitative data was supplemented with prospective data collected during one month. Semi-structured interviews, focus group discussions (FGD) and informal observations served as the sources of data on the qualitative end.

**Results:**

In total, 22 % of maternity clients refused CS and more than 90 % of the CSs in the focal hospital were emergencies which may indicate late arrival at the hospital after seeking assistance elsewhere. The qualitative analysis reveals that socio-cultural meanings informed by gender and religious ideologies, the relational consequences of having a C-section, and the role of alternative providers are some key factors which influence when, where and whether women will accept C-section or not.

**Conclusion:**

There is need to find means of facilitating necessary CS by addressing the prevailing socio-cultural norms and expectations that hinder its acceptance. Engaging and guiding alternative providers (traditional birth attendants and faith healers) who wield much power in their communities, will be important to minimize delays and improve cultural acceptability of CS.

## Background

Maternal mortality remains a serious problem in low and middle income countries (LMICs) which account for 99 % of the global number of maternal deaths (284 000). Sub-Saharan Africa alone accounts for 56%of the global burden [1]. Maternal deaths are clustered around labour, delivery and immediate post-delivery periods [2]. Key contributory factors in LMICs, including Nigeria, are delays in receiving obstetric care [2].

The Millennium Development Goal 5 (MDG-5) aims to reduce maternal mortality by 75 % by 2015 and, of the African countries that are associated with high maternal deaths, very few of them are on track [1]. The global community has invested substantial resources in maternal health programmes, and countries in Africa including Nigeria have responded with strategic policies. However, the results fall below expectations. Nigeria’s Maternal Mortality Ratio (MMR) has gone down from 1100 in 1990 to 563 in 2014, but falls clearly short of its MDG-5 target of 300 [3]. The importance of continued effort to reduce maternal mortality is acknowledged by its inclusion in the Sustainable Development goals [4].

The World Health Organisation [5] identifies the availability of skilled birth attendants (SBA) and provision of Emergency Obstetric Care (EmOC) as two of the most essential ingredients of maternal mortality reduction programmes. EmOC can be basic or comprehensive (bEmOC or cEmOC) depending on the constituent signal functions. Caesarean section (CS) is a component of cEmOC and stakeholders agree that it should be universally available and accessible [5]. Major indications for CS in Africa include abruption placentae, previous CS, foetal distress, malpresentation, preeclampsia/eclampsia, placenta praevia, prevention of HIV infection in the new born and, most commonly, obstructed labour [6, 7]. In the absence of CS, obstructed labour may result in major peri-natal and obstetric complications which greatly affect quality of life, including vesico-vaginal fistula (VVF), recto-vaginal fistula (RVF), and stress incontinence. Ultimately, obstructed labour can result in death of the mother and baby [8].

The optimum CS rate, which is the estimated proportion of deliveries in a population that will need CS, ranges from 5 % to 15 % [5, 6]. Some facilities in Nigeria report CS rates within [9] or even above [10] the estimated optimum range, the latter leading to concerns about CS over-use. However, at a national level, the CS rate of about 2 % [11] is far below the expected optimal minimum. Similarly, low CS rates of between 1-2 % can be found elsewhere in sub-Saharan Africa, including West Africa [12, 6]. Moreover, there is great inequity in access to CS based on economic capacity; in Nigeria rates drop below 1 % for the poorest 80 % of the population [13]. Low CS rates are indicative of unmet obstetric need for potentially life-saving care and appear to be an important contributor to perinatal mortality as well as maternal morbidity and mortality [14, 15]. Indeed, Dumont and colleagues [6] argue that ‘one of the most effective means of reducing maternal mortality is the provision of CS for all women who need them’(p. 1328).

The problem is not only low CS rates, but also high rates of emergency CS. An earlier study in Nigeria [16] found that emergency CS accounted for more than 80 % of the operations over a 16 month period. Given that elective operations tend to have better maternal and foetal outcomes than emergency CS,an increase in *planned* and *timely* CS is desirable [15–18]. Provision of cEmOc, including CS, can best reduce maternal mortality if women access facilities on time. Delays in accessing maternal health services can occur in different phases [19]. Phase 1 delays pertain to the decision to seek ‘appropriate’ medical care on time on the part of the individual, family (including spouse) or both. Phase 2 relates to delays in reaching an appropriate healthcare facility. Phase 3 concerns delays in receiving adequate care at the facility. In relation to CS, phase 1 delays appear particularly relevant. Several Nigerian studies have reported on women’s aversion regarding the procedure which islikely to lead to delays or refusal of CS [20–24]. Aversion appears grounded in fears that C-section results in health complications such as infertility, or even death [22, 24], and also in socio-cultural meanings attached to C-section [21, 22].

**Fig. 1:**
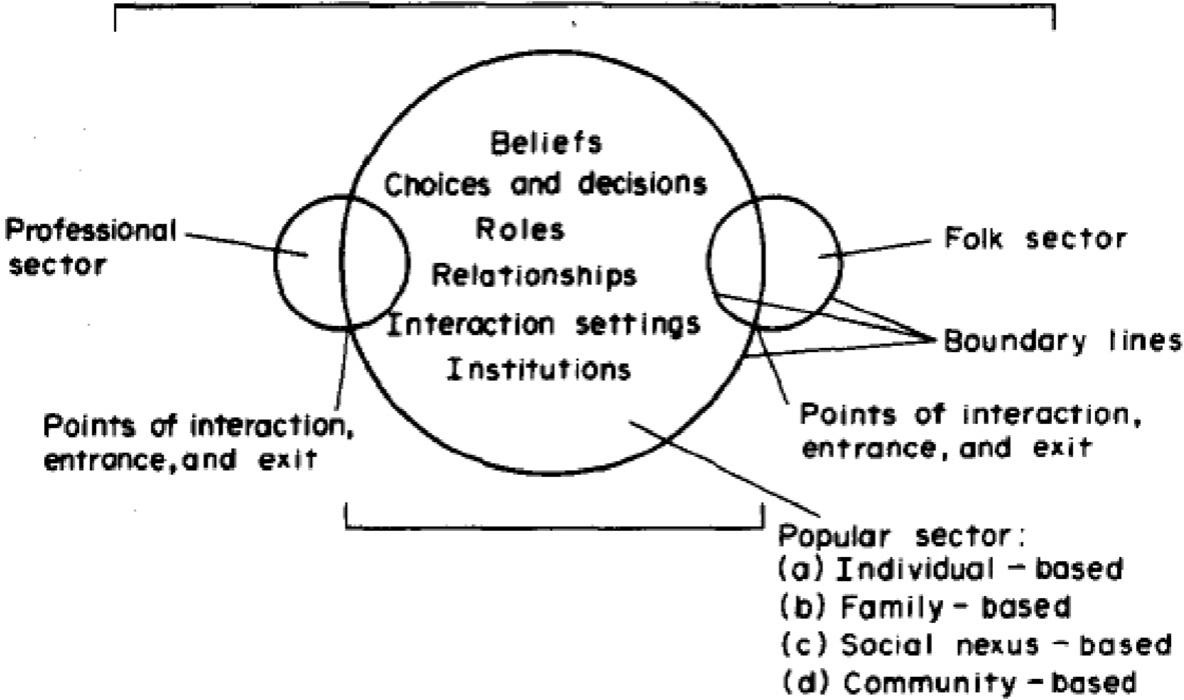
Kleinman’s Explanatory Model

In this paper, we seek to further our understanding of how the socio-cultural context influences uptake, or rather refusal, of C-Sections. We draw on anthropologist Kleinman’s classic arguments that medical systems are cultural systems, like kinship and religious systems, and intertwined with meanings, values and behavioural norms [25, 26]. Moreover, medical systems encompass multiple arenas within which people experience and manage sickness. Many health problems are resolved in the ‘popular arena’: the family and social networks. The professional arena consists of professionalized biomedicine; the folk arena includes non-biomedical healers including traditional birth attendants (TBAs) and spiritual, faith-based providers. These arenas uphold different beliefs, expectations, roles and relationships (see Fig. 1) and are characterized by different explanatory models (EMs), or social constructions. EMs are context-bound sets of ideas about aetiology, onset of symptoms, pathophysiology, course of illness and appropriate treatment. When EMs in the professional and popular arena differ, biomedical professionals may cure a ‘disease’ - malfunctioning biological and physical processes - but not ‘illness’, that is, the experience and societal reaction to (perceived) disease [25]. Thus, biomedical practitioners may cure the biological dimensions of obstetric complications (‘disease’), for instance through CS, but not ‘obstetric illness’; the experience and social reactions that ultimately determine health-seeking behaviour. Folk healers’ success in communities may be based on their ability to cure illness rather than disease. They can provide cultural healing, by offering personally and socially meaningful interpretations of the illness experience, in part because their explanatory model resembles the community’s more closely [26].

It is important to note that, as Kleinman and other anthropologists have pointed out, biomedicine too, is a cultural system like’folk healing’. Anthropologists of reproduction have highlighted how the biomedical model of birth, although ostensibly derived and grounded in objective scientific ‘facts’, expresses particular cultural values [27]. Feminist anthropologists in particular have critiqued it for being ‘technocratic’, over-emphasizing technology and medical risks at the expense of a more holistic, naturalistic approach to birth which acknowledges the woman’s experience and allows her rather than the medical practitioner to be in control [27]. Differences between biomedical and ‘lay’ models of birth are not absolute. For instance, Makhlouf Obermeyer describes how in Morocco community members acknowledge health risks but refrain from taking preventative action because of practical barriers or because other risks such as receiving more disrespectful care are deemed more important [28].

With regard to pregnancy complications and CS, we can note a mismatch between professional, folk and popular models. Whilst CS is seen as a highly appropriate intervention in the professional, biomedical arena, it may be seen as ‘reproductive function failure’ on part of the woman in the popular sector (i.e. communities) and a desire to experience vaginal delivery can become reason for CS refusal [15, 24]. Thus, whilst the biomedical explanatory model considers CS appropriate treatment, the prevailing cultural model does not.

In addition to explanatory models, gender roles and relations need to be acknowledged to fully explain the influence of socio-cultural values on CS refusal. Within society, people are assigned expected attributes, behaviors, and responsibilities based on being a male or female [29, 30]. These gender roles are social constructions, which differ according to the specific socio-cultural, economic and historical context [31]. Nigerian traditional society is characterized by patriarchy, that is, it is a society in which men tend to hold the positions of power, and characterized by social stratification on the basis of sex [32]. The social system fosters gender inequalities by relegating women to domestic and reproductive roles and restricting their access to finance, and other entitlements such as land [32]. Hence, patriarchy and the social construction of gender roles in Nigeria –and many other countries - constrain women’s autonomy and access to resources [33]. These gendered socio-cultural arrangements also limit women’s capacity to make health related decisions, including their capacity to accept CS, thus increasing their risk of potentially life-threatening pregnancy complications [34]. The prevailing socio-cultural pressures to achieve vaginal delivery are likely to be a driving force behind some Nigerian women’s use of additional providers in an attempt to achieve ‘normal’ (vaginal) childbirth and avoid CS [35]. For instance, some women in Nigeria have been observed to make multiple ANC bookings, and this may in part be a ‘back-up plan’: if one provider advises CS, women turn to another, hoping for a different pronouncement [35, 36]. In addition, some may stick to the ‘popular’ arena, giving birth at home while others opt for alternative ‘folk’ providers such as traditional birth attendants (TBAs). The 2013 Nigeria Demographic and Health Survey (DHS) [11] reports that TBAs attend as much as 22 % of births. Characteristically, TBAs are usually older women with inherited childbirthing skills and tend to practice in their local communities. Many of them do not have designated delivery rooms and their clients are usually women of relatively low socio-economic and educational status [37]. While factors such as distribution of facilities, cost and quality of services are all relevant influences on women’s choice of place of birth, some Nigerian women appear to prefer TBAs because they are said to ‘never cut any woman open’; that is they are not associated with CS [21, 24, 38]. Furthermore, *religious* providers seem to be re-shaping the ANC and delivery landscape by promising outcomes based on ‘faith’ and ‘divine protection’ rather than on child birthing skills [39, 40]. Religious providers represent a diverse group of faith-based outlets ranging from birthing outlets linked to established churches and mosques to stand-alone small spiritual homes owned by individuals. These faith-based providers share a common feature of promising good delivery outcomes derived from divine/supernatural involvement. According to the Nigeria DHS [11], more than 98 % of the survey respondents identified with either Christianity or Islam, making Nigeria a deeply religious country. The religious environment together with socio-cultural, gendered pressures on women may drive women to religion-based birthing centres, mostly churches, in part out of hope that a divine or supernatural intervention will lead to a vaginal delivery [41]. There are risks attached to giving birth with these alternative providers. TBAs may have a role to play in low income settings with limited human resources for health [42] but are unlikely to have full knowledge for instance about HIV [43], and thus may not take necessary preventative measures. More so, TBAs cannot provide EmOC, nor can religious providers and some evidence suggests that birth with religious providers isparticularly risky. For example, Etuk et al. [39] found that pregnancy outcomes in churches were worse compared to deliveries conducted by TBAs: all the maternal deaths recorded in the study occurred in the church. Using TBAs or religious providers will be particularly risky and indeed potentially lethal for pregnant women whose condition would necessitate CS. Their fate will depend, in part, on the alternative providers’ recognition that they indeed need the procedure and their willingness to refer to an appropriate facility.

This paper further illuminates the gendered, religious and socio-cultural drivers underpinning CS refusal and use of alternative providers, by reporting on a mixed-methods case-study conducted in a missionary hospital in North-Central Nigeria. The study’s research questions were:

1. What is the prevalence of CS (and emergency CS) in a missionary hospital?
2. How often do women decline CS in this hospital?
3. How may socio-cultural issues contribute to delays and under-utilization of CS in this community?

## Methods

### Design

**Fig. 2:**
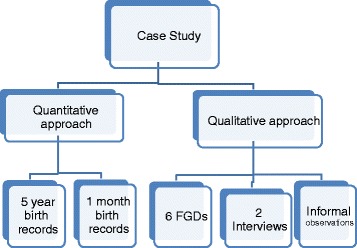
Schematic representation of the study design

In this small-scale study we adopted a sequential mixed methods design (see Fig. 2) so that we could use the findings of one (qualitative) method to elaborate on the findings of another (quantitative) method [44].

### Setting

The study setting was a Missionary health institution in the North-central region. It is a secondary health facilitythat has basic medical and surgical departments. Whilst located in a semi-urban area, the community represents a mixture of urban and rural lifestyle due to the presence of a university. The community is thought to exist out of equal numbers of Christians and Muslims. In addition, a small number of people can be said to be traditionalists; they describe them as people who align themselves with the cultural practices and the custom of the land.^1^ Polygamy seems common among the Muslims and traditionalists.

The Queen Margaret University (QMU) Ethics committee and the Hospital board both granted ethical approval. The study was undertaken from June 1, 2012 to July 1, 2012. The data for quantitative analysis came from a five year (Jan. 2006-Dec. 2010) retrospective review of hospital records concerning method of delivery and cases of CS refusal. However, because the records did not have sufficient information on patients declining CS and sometimes did not differentiate between emergency and elective CS, prospective data on the same subject was collected for the month of June, 2012.

There were two major sources of qualitative data in the study: semi-structured interviews and focus group discussions (FGDs). The interviews provided in-depth views of women who had undergone CS in the community while the FGDs explored norms, meanings and socio-cultural values and how they affect decisions relating to CS [45]. Informal observations by the first author were written up in field notes.

### Participants and sampling

Two key informant interviews with post-Cs women and six FGDs with four or five participants were held. Out of the twenty-nine participants (2 in interviews and 27 in FGDs) 21 were female. Men participated in the FGDs with fathers (4 men) and providers (4 men). The female perspective was thus over-represented. The FGDs were constituted thus:

- Ante-natal care (ANC) clients (2 FGDs) represented the views of expectant mothers who could be confronted with the CS option.
- Mothers in a post-natal and under-five clinic (1 FGD) represented women with a birth experience (either vaginal or CS).
- Fathers (1 FGD): representing the male perspective on the issue, important considering their influence on care-seeking behaviour.
- Nurse/Midwives (1 FGD): Nurse-midwives are usually the first contact of the women during delivery and may have valuable insights on dynamics between patients and their families.
- Providers (1 FGD): comprised of senior hospital staff with both clinical and administrative positions.

All participants were recruited within the hospital premises. Due to the sensitive nature of the subject of inquiry and the cost and time restrictions, convenience sampling was employed for both FGDs and SSIs. This problematizes generalizing the findings, but findings from convenience samples can serve as springboard for further research and can be linked to other existing findings reported in the literature [46]. Inclusion criteria specified that participants had to be married, over 18 years (the legal adult age in Nigeria) and speak English or Pidgin^2^ English. We excluded the following more vulnerable people from participation; women who suffered significant childbirth-related complications or had lost a baby in the last six months, or without children or a partner/husband.

### Study procedure

The first author conducted the interviews and the FGDs. They took approximately 45 min and were conducted in quiet hospital rooms and were all digitally recorded. Recordings were transcribed verbatim and to enhance the reliability of findings, the transcripts were re-checked carefully for mistakes. Reflexivity is important in qualitative research. We had planned to use a female research assistant fluent in the local language to conduct some of the FGDs, but did not find a suitable assistant. The first author conducted the interviews and FGDs in mostly Pidgin English. This will have limited the contributions of some less educated or rural inhabitants who may not feel comfortable in these languages.

### Data analysis

Descriptive statistics of the five-year delivery data were calculated with special emphasis on the yearly CS rates and the five-year average. The same was done for the prospective data collected for June 2012 which includes categorization into elective and emergency CS and the number of women who refused CS in the month under examination.

Thematic analysis was employed for interpretation of the transcribed qualitative data. This entailed reading the data repeatedly until patterns were identified and classified into themes and sub-themes. Both descriptive themes and analytical themes were identified and coded. Descriptive themes refer to tangible and easily identifiable aspects that may not need much interpretation while analytical themes relate to more abstract, theoretical notions that require inference on the part of the researcher [47].

### Ethical considerations

Study participants received oral and written information in local language regarding the nature and purpose of the study. Participants were reminded and reassured that they were under no obligation to partake in the exercise, and that refusing to participate would not affect their access to hospital services. They were informed that they could decline to comment on any issue or withdraw from the exercise at any moment they so desired without having to necessarily offer any explanations. Written consent was obtained from all the participants who met the inclusion criteria before the study process commenced. Confidentiality of all information and anonymity of all statements/persons was upheld by removing the participants’ names and replacing them with codes. Soft data was stored in a password-locked computer and hard copies were kept in a secured box to which only the researcher had access.

## Results

### Quantitative findings

Analysis of the 5 year records (2006–2010) show that out of a total of 5353 deliveries,4611 were through vaginal delivery and CS accounted for742 (Table 1). The indications for the C-sections were mainly prolonged/obstructed Labour, foetal distress, preeclampsia/eclampsia, ante-partum hemorrhage consistent with standard medical indications for CS.

**Table 1:** Delivery data

YEAR	Vaginal delivery	CS	EmergencyCS	CS refusal	Yearly Total
2006	704	118	ND^a^	ND	822
2007	963	154	ND	ND	1117
2008	1080	179	ND	ND	1259
2009	987	155	ND	ND	1142
2010	877	136	ND	ND	1013
5-year total	4611	742	ND	ND	5353
Yearly average deliveries	922	148	ND	ND	1071
Monthly average	77	12	ND	ND	89

^a^ND implies ‘No Data’

This translates into a monthly average of 89 deliveries and 12 CSs. Thus, CS accounted for about 14 % (see Table 2) of all deliveries over this period.

**Table 2:** Delivery data from June 1, 2012 – June 30, 2012

Vaginal delivery	83
CS (total)	14
Emergency CS	13
Elective CS	1
CS refusal	4
Total deliveries	97

According to the prospective data, the hospital recorded a total of 97 deliveries in June 2012 (Table 2). There were 14 CSs; 13 emergency CSs and only one elective CS. In the same month, four women who had been medically booked to have a CS declined the surgery and left the hospital.

The combined data-sets indicate that CSs accounted for about 14 % of all deliveries out of which more than 90 % were emergency operations (Table 3). Based on the data for June 2012, four (4) women refused to undergo C-section and left the hospital for various reasons. Although this constitutes a small proportion (4 %) of the total number of deliveries (97) and 22 % of all medically indicated CS, it confirms that some women do refuse C-section and that this occurs on a somewhat regular basis. The period covered by the prospective data collection was relatively short, but the month of June 2012 seems representative in terms of total deliveries, vaginal deliveries and CSs (Table 3). The sample size is small and does not allow for firm conclusions, but paints a picture of birth practices in our focal hospital.

**Table 3:** Side-by-side view of the 5 year birth records and the 1 month records

Variables	Monthly average between Jan.2006 – Dec.2010	June 1 – June 30 (2012)
Total deliveries	90	97
Vaginal deliveries	76	83
CS deliveries	12	14
Emergency CS	ND	13
CS refusal	ND	4
CS as a percentage of total deliveries	14 %	14 %
Emergency CS as percentage of total CS	ND	93 %
CS refusal as a percentage of total medically indicatedCS	ND	22 %

### Qualitative findings

Main findings are presented according to the following broad themes: cultural perceptions, gender roles and religion, post-CS social consequences and influence of alternative providers.

### Cultural perceptions, gender roles and religion

Cultural understandings shape people’s perception of a health problem [25]. The data reveal a perception of vaginal delivery as what has traditionally happened, and the normal form of delivery, expected of every ‘proper’ woman. See extract 1.

#### Extract 1

*“…as a woman, naturally they should be able to carry the pregnancy and have a normal delivery, that is what is expected. That their parents in the time past went through these rigors of labour and delivered without surgical intervention, and so they see no reason why they cannot deliver…” (FGD8-providers)*

The participant in extract 1 suggests that the construction of vaginal delivery as natural, normal delivery is grounded in past experiences. However, only some experiences are made relevant: parents delivering without intervention, but not the fact that presumably some of them died, were left with disabilities or lost their babies in the process. This construction of vaginal delivery as normal and natural may constitute a barrier to accepting other methods of birth, as participant in extract 2 indicates.

#### Extract 2

(P4*)” …most of them that come and have the surgery have tried [other methods and providers and they failed] before they come. There is nothing like elective surgery [CS] here…”(FGD8-Providers)*

The construction of vaginal delivery as normal, natural and preferred means of delivery appears linked to the social construction of gender and gender roles; vaginal delivery appears to be a symbol of womanhood. Consider extracts 3 to 5.

#### Extract 3

*(P2)“…our people believe that if you don’t deliver through the vagina, you are not a woman. So they have that in mind when they are with you [the doctor]…” (FGD8-providers)*

#### Extract 4

*“… I like to have natural delivery like a “Woman” and I know a lot of other women like that…” (IW2-Post-CS client)*

#### Extract 5

*“in my area, you don’t become a woman until you have a successful vaginal delivery. This is because you are considered a woman… until after you have experienced and endured labour pains” (Others nod)(FGD7-Nurse/Midwife)*

The respondents, including providers and a woman who had undergone CS refer to a shared idea that ‘true women’ give birth through vaginal delivery. Thus, vaginal delivery plays a gate keeping role to being described as a ‘true’ or ‘proper’ woman. This construction is likely to limit consideration of risks, as seen in extract 1. This is problematic especially if a woman has been advised against vaginal delivery. Other data also suggests that there may be limited appreciation of risks to the health of the mother or baby as reasons for CS. When asked what they understood or knew about CS, several respondents, practitioners and community members alike, focused on the inability of a woman to deliver on her own:

#### Extract 6

*“… the operation they carry out on a pregnant woman who cannot deliver on her own and the only way is by the operation” (FGD5-Mothers)*

#### Extract 7

*“A form of assisted delivery that takes place when a woman cannot deliver on her own and she is taken to operating theatre…” (FGD7-Nurse/Midwives)*

Emphasis on inability to deliver on one’s own de-emphasizes considerations such as risks for the mother and baby. For instance, a HIV-positive woman may be advised to have a CS as part of Prevention of Mother To Child Transmission (PMTCT) strategy in which case CS has nothing to do with ‘ability’ to achieve vaginal delivery. Describing the essence of CS with phrases like’cannot deliver on her own’ both reflects and reproduces associations with lack of strength and even laziness :*“sometimes they do look at them as lazy women” [FGDx-Mothers] .*

Thus, having had a CS may lead to perceptions that one is an un-able, weak or lazy woman. These are socio-cultural consequences which women would want to avoid, for instance by refusing CS.Another way in which social-cultural perceptions and gender roles become barriers is that CS is perceived as placing a restrictive ceiling on the possible number of children a woman can have, as some of the women attending ANC indicated.

#### Extract 8

*“…we have heard that after the first and second CS operation, it will be difficult to give birth to the third child. Because of that if a couple plans to have 5–6 children and in between you recommend a CS the woman will say no” (FGD4-ANC2).*

#### Extract 9

*“This CS operation is not even advisable, because if you give birth through CS for the first and second time. They will now be recommending that you give birth through CS and you won’t be allowed to have the number of children you want to have. So the CS operation is very risky” (FGD4-ANC2).*

The concern that CS prevents one from attaining one’s ideal family size is pertinent especially in a high fertility context; the 2013 DHS indicates that Nigeria’s total fertility rate is 6.0 [11]. Consequently, women who have been advised to have CS may well opt for alternative treatment. Since alternative providers like TBAs or faith-based homes conduct only culturally acceptable ‘normal’ delivery, women may use their services in pursuit of social conformity and out of desire to fulfill societal gender roles.

Moreover, P4 in extract 1 indicated that CS is only considered if all other options failed. This is particularly likely in contexts where many believe that problems with pregnancy and delivery that culminate in the need for CS can be a result of a curse or spiritual attack and therefore require folk healers’ interventions. The following extracts express some participants’ views on the possible reasons why a woman may need a CS:

#### Extract 10

*P1 “some believe that it is a curse and the problem is spiritual: some believe that their second*^3^*or an enemy may be responsible for the problem” P2 “- I once overheard my neighbour’s mother –in-law telling a friend that if her daughter-in-law misbehaves again, she will ensure that the daughter-in-law undergoes another CS operation” (FGD3-ANC clients).*

#### Extract 11

*“Yes, some will say that your enemies locked your waist with a padlock that is why you couldn’t deliver naturally unless through CS” (FGD4-ANC2).*

As Kleinman [25, 26] suggested, different arenas in medical systems yield different explanations of the nature of the problem and remedial action required and people may visit folk practitioners because they offer culturally meaningful explanations. Beliefs that an enemy could attack someone spiritually and impose punishments such as inability to have vaginal delivery may well affect women’s health seeking behaviour when faced with delivery complications. In such a case, many women and their families would believe that traditional healers or religious priests are better able to deal with it than the hospital.

There is another reason for the patronage of religious providers to avoid CS: it can become a demonstration of faith. It is believed that CS can be avoided by ‘emphasizing your faith’ in God; through divine intervention, vaginal delivery will be possible. Conversely, a CS could cast doubt on one’s religious status. A participant drew evidence from the bible –thus emphasizing her own religiosity- to bring this viewpoint to the fore:

#### Extract 12

*“God told us that if we tell this mountain to move it will be moved. CS is not a normal portion for a believing woman; it is in abnormal cases that you see CS” (FGD5-Mothers)*

A ‘believing’ woman refers to a Christian faithful. There thus seem to be two cultural understandings relevant for choice of alternative providers: supernatural causes may lead to complications which, from a medical perspective, necessitate CS; and divine intervention can help a woman avoid this intervention. According to both lines of thought, asserting ones faith can help achieve vaginal delivery. Women who do accept CS may therefore be viewed as not ‘strong’ enough as a woman but also in terms of faith. To avoid doubts about one’s faith, women may refuse CS. CS may have other social consequences; it may affect women’s relationship as we discuss t in the next section.

### Relational consequences of CS

As discussed, preferred mode of delivery appears related to gender roles; ‘proper’ Nigerian women have a vaginal delivery. Not surprisingly, CSs may have relational consequences, and these may deter women from consenting to the procedure. A provider illustrates this view:

#### Extract 13

*“People have a certain number of children they want to have. They have the belief that once you are operated you will continuously be having CS and this will reduce the number of children they can have…once you are married and you go through CS, the husband may decide that this lady is not competent enough to give him the desired number of children so he may even decide to marry another wife that can have vaginal delivery” (FGD8-Providers).*

This extract demonstrates how being seen as ‘incompetent’ in terms of having a vaginal delivery and bearing many children, jeopardizes one’s status as wife; the husband and family in-law may bring an additional ‘competent woman’ into the marriage. Some women may not even have the ‘option’ of sharing the husband with another woman as she may be abandoned completely after the surgery as illustrated in the extracts below:

#### Extract 14

*“In fact, there was this patient who had CS twice. When she got pregnant the third time the mother-in-law called her and told her that if she does not look for a way to deliver that baby by herself and she ends up with a CS, that she will be going back to her father’s house from the hospital bed- that her son won’t be marrying her again as she keeps wasting her son’s money on CS when other women are giving birth by themselves.*

*Unfortunately for the woman, she still ended up with a CS and that brought problems for her right from the hospital. The husband, who happens to be fairly economically stable, married another wife almost immediately” (FGD3-ANC1)*

#### Extract 15

*“In this hospital, some husbands have abandoned their wives on account of having had a CS. They leave them because they can’t afford to pay for the hospital bill” (FGD6- Fathers)*

Thus, CS may disrupt a woman’s marriage, and the costs of the surgery appear one factor which can disrupt marriage . As one woman who had undergone Caesarian section explained:

#### Extract 16

*“…he [the husband] takes every little opportunity to remind me that he spent so much money in ordinary childbirth and that he won’t do that again, as if he alone paid all the hospital bills” (IW2-post-CS)*

Conversely, a father asserted “*we consider women who deliver through vagina as economically safe” (*FGD6-Fathers*).* Kleinman et al. [26] note that social and economic status influence clinical realities. It may be especially difficult for women of low socio-economic status to accept CS deliveries, as evidenced by socio-economic differentials in CS rates reported in the literature [13]. However, extract 14 indicates that also women married to husbands who *are ‘fairly economically stable’* may encounter marital trouble. Furthermore,the statement ‘*look for a way to deliver that baby by herself’* in extract 14 suggests that there is a belief that a woman has control over her method of delivery, reflected as well by the aforementioned view that women who have undergone CS are lazy. Thus, husbands and in laws may hold a woman responsible for having to undergo a CS and this may reduce their sympathy. Hence, a woman may be pressured to find means of having a vaginal delivery, irrespective of consequences to her health and that of the baby.

Extract 14 makes clear how in-laws can contribute to marital trouble. Other family members may also affect the post CS experience. In particular, being in a polygamous marriage may affect the dynamics as seen in the extract below:

#### Extract 17

“*I have these neighbours-two women married to one husband- the first wife had all her children through CS and the second has been having successive vaginal delivery. The second wife taunts and oppresses the first wife by making comments like, ‘I have enough strength like a woman and has been pushing my babies out by myself while the people that don’t have strength are being ‘cut’ like a meat every time’” (FGD3-ANC1)*

It appears that women who have had a CS may be abused by co-wives. In the example of a taunting statement ‘*I have enough strength like a woman’, we see again* the notion that women who have CSs are ‘weak’ and ‘unfit’ to be women. This will be worrisome for both a post-CS woman and those advised to undergo CS alike. Women may thus refuse CS for fear of failing in their culturally assigned gender role, which may endanger their marriage. It may therefore be particularly difficult for primigravidas to accept a C-section. Having undergone a previous vaginal delivery may lessen the socio-cultural pressure on a woman; she has already proved herself capable of achieving vaginal delivery. When asked if she would accept another CS, a post-CS woman said:

#### Extract 18

*“God forbid! Apart from the monetary involvement, I really don’t like it. In fact, I only consented to this one because it wasn’t my first childbirth” (IW2-post-CS).*

Asked what she would do if it happened to be her first delivery, she said:

#### Extract 19

*“No! Do you know what people would say. My husband’s family would have seriously gotten involved- and that would be bad for me” (IW2-post-CS).*

Given the profound social consequences, it is not surprising that CS seems surrounded with much fear and that those who eventually accept CS have often tried other alternatives during labour. CS is then accepted only as a last option and thus, is mostly carried out as an emergency surgery (see Tables 2 and 3).

### Alternative providers

Beliefs and labels given to a health problem inform what remedies are considered appropriate and meaningful [25, 26]. The practices of alternative providers seem to be culturally meaningful and accepted by many and alternative providers appear preferred whenever the reason for CS is interpreted in the light of a ‘curse’, ‘enemy attack’ and/or ‘spiritualism’. Moreover, participants identified fears associated with pregnancy and delivery as a factor in the use of alternative providers, some women may seek their protection. Whilst many religious denominations appreciate biomedicine and the medical need for CS, some religious leaders do not. So religious leaders may advise women against CS, fueling the aversion and delays by promising that an alternative method based on faith and prayers can achieve vaginal delivery. As one provider explained:

#### Extract 20

*“It depends on the religion- some elite churches like catholic who believe on modern things know that there is room for education and that God uses us to do miracles. But for some other people who believe that you can pray and this building will just move to the next place, they are the people creating the problem.…You know that religion has so much roles in our lives here that if you don’t believe they will bondage you that you are going to hell for not believing that God can do anything. They tell you things like, ‘woman you are going to deliver naturally if the doctor says otherwise that doctor is a liar’. Of course, that person will believe and that’s what she wants to hear…If she goes to another church maybe a catholic church and the priest is not able to tell her that, a friend in the neighborhood will say, ‘why don’t you try this church?’ and like that she continues going to all the churches until she will see a pastor who will say ‘you will deliver naturally without CS’ and that’s what she wants to hear.” (FGD 8- Providers).*

This account suggests that some religious leaders’ position themselves as people who can ‘spiritually’ guide women to reach the social goal of achieving vaginal delivery, even against medical advice. The reference to ‘*that is what she wants to hear’* (Ex. 20) highlights how religious providers’ influence ‘feeds off’ socio-cultural preferences, understandings and norms.

Similarly, many TBAs would refer the woman to the hospital when necessary but “*some of the TBAs try to prove that they are better than the hospitals and that they can take any delivery (FGD6-Fathers).* Some participants acknowledged that TBAs perform essential functions at the community level, and have the potential to guide the people in making reasonable decisions with regard to CS. However, it was suggested that some may wait until the last minute before taking appropriate steps. One provider comments on TBAs:

#### Extract 21

*“The issue is that if the woman is with them before hospital, they delay them. And then again, if the woman is close to them [in terms of proximity] while in the hospital, maybe in cases of prolonged labour and you ask them to consent for CS, they will first go there to consult them. If they [TBAs] permit, you go ahead and do the CS, if they don’t permit, you won’t do the surgery. And again, they send them their concoctions- they send them to a woman in labour while in the hospital and say, ‘drink, you will deliver’. It is almost the same thing with pastors, ‘you will deliver before surgery’. And in some cases, they even run away from the hospital to them as well.” (FGD8-Providers)*

This extract suggests that alternative providers have substantial power over their clients; they may decide who would have a CS and who should not. This consent process and promises of vaginal delivery will lead to delays in uptake of CS. The reference to women consulting alternative providers while in the hospital (extract 21) appears to reflect the alternative providers power within the community, society’s trust in them, and the degree of pressure women face to avoid CS.

## Discussion

We established a C-section rate of approximately 14 % in our study site. Emergency CS accounted for more than 90 % of all c-sections, which suggests that delays occurred in one or several phases of the care seeking process. More so, 22 % of medically indicated C-sections were refused by the women. This finding was supported by the qualitative data; several respondents spoke of the possibility that women refuse C-section although we were unable to collect first-hand accounts of Cc-section refusal. Furthermore, the qualitative analysis reveals that socio-cultural meanings, informed by gender and religious ideologies, the impact of C-section on marital and household relationships, and the presence of powerful alternative providers are key factors which influence whether and when women will accept C-section or not. These socio-cultural and religious, gendered influences may well lead to delays and emergency operations, reported as common in other Nigerian studies as well [16].

The study setting is a longstanding missionary hospital with a good reputation in terms of care and affordable and flexible payment. It is thus likely to be seen as a preferred place for CS and, as such, to have higher CS rates than many other facilities. For the same reason, rates of C-section refusal may well be higher in some other hospitals. Very few studies report CS refusal rates. One other Nigerian study found a much lower refusal rate of 11.6 % [22]. This study was however, conducted in a teaching hospital in a wealthier and more urbanized setting. The literature reports a positive association between socioeconomic status and CS rates [12, 13]. This will largely be due to increased access, but we can also expect that in low and middle income settings, women of higher socio-economic class have less aversion to CS [20]. Our qualitative findings also indicate that costs contribute to women’s aversion for CS in part because when a woman or her husband cannot easily pay for her CS, chances are that her marriage may also be threatened. This echoes findings from a previous study conducted in Burkina Faso, which found that obstetric emergencies can lead to marriage breakdown, in part due to costs incurred [48].

Our findings support other scholars’ claims regarding the importance of acknowledging socio-cultural interpretations [26] *and* gender norms and power-relationships [29, 31, 33, 49] in the pursuit for meaningful health interventions. Consulting a faith healer when advised to have a CS may be grounded in local explanatory models, such as the idea that one is cursed [41], but gendered, normative expectations that ‘proper’ women give birth ‘naturally’ matter too. In our study setting, the risk of being seen as a ‘failed’ woman and wife appears to contribute to CS refusal. As Okojie [34] maintains, socio-cultural constructions which emphasize women’s childbearing role contribute to gender inequalities in health and disempower women.

Restrictive gender roles are reproduced in interactions between women and their spouses, but mothers-in-law are also important [49]. They may possess much power over their son’s wife because men see birth as the domain of women and do not usually get involved in the process [50]. Also, mothers-in-law may influence ‘family finance’ [49]; we have seen how the pressure from a husband *and* relatives to achieve vaginal delivery seem related at least in part to CS associated costs. A woman’s access to resources, including those needed for CS may depend on the quality of her relationship with her mother in law [49]. These complex socio-cultural entanglements make acceptance of C- section difficult, and if women are to consent to CS, it would have to be the last option.

It appears then, that reducing maternal mortality in Nigeria will require more than making EmOC available [41]. We concur with Brunson [50] (2010, p.1725) that “local acceptance of biomedical system of knowledge does not necessarily lead to the utilization of services in a timely manner if neither women nor men are in culturally-defined positions to act”. Women’s limited capacity to act emanating from gender and other socio-cultural concerns is potentially life threatening. Some communities in Nigeria, and perhaps in other low and middle income settings, see CS as an option that can only be subscribed to, if at all, when other alternatives have failed, leading to high proportions of emergency procedures. Thus, socio-cultural, gendered ideals contribute to delays and we can expect that this will affect maternal morbidity and mortality.

The role of alternative (traditional and religious) providers is important. They appear to benefit from the social pressure on women to have vaginal delivery and may contribute to delays when they send women late to the hospital. More so, alternative providers may directly or indirectly contribute to women’s reluctance to have a CS; their guarantees of vaginal delivery may make women doubt the biomedical advice that CS is necessary. Furthermore, alternative providers foster belief in supernatural causes of a woman’s inability to achieve a vaginal delivery by positioning themselves as people who are able to address these ‘root causes’. Hence, having been placed in a position of power by the prevailing socio-cultural dynamics and belief system in the community, alternative providers may reinforce aversion to CS and contribute to delays in women accessing CS. However, it is important that future research gains more direct access to alternative providers’ practices and views; we had to rely on providers’ and community members’ accounts (see Limitations).

We noted that the reported aversion against CS co-occurs with concerns about over-use of CS in some Nigerian facilities [10]. The medicalisation of childbirth has been problematized by feminist anthropologists for decades [27]. Critique has centred on how medical interventions shift control from the woman to the often male medical practitioner, and the medical model’s prioritization of risks to health over and above social and personal risks [28]. As such, women’s refusal of CS could be seen as rejection of medical ‘authoritative knowledge’ and display of agency, understandable within the specific socio-cultural context [51]. It is however important to note the potential detrimental consequences to women’s health and well-being. Furthermore, women who walk away when advised to undergo CS appear to exert agency within restrictive circumstances confined by prevailing socio-cultural norms and gendered vulnerabilities, characteristic for socio-economic contexts where women’s economic and social survival may depend on their marriage and thus reproductive potential [48].

### Study limitations and recommendations

We need to acknowledge several limitations. We planned to use a local female research assistant in recognition of gender concerns and language barriers. Due to financial and time limitations no suitable assistant was found and the first male author conducted the interviews and FGDs. This excluded those who did not speak English and may have affected participants’ responses. However, most participants seemed to feel at ease and spoke freely. Furthermore, because the research was conducted in one community, and involved a modest number of participants, we must be cautious about the generalizability of the findings, especially considering that Nigeria is a multi-cultural society with various ethnic groups. In fact, the perspectives of the participants in the hospital may be different from that of the host community since both the background of the staff and the users of the hospital extend beyond the boundaries of the community. Nonetheless, given similarities with findings reported in other studies, some of our findings appear to reflect wider socio-cultural ideologies that are seen to be at odds with CS. Another major limitation of the study hinges on reliance on the perspectives of biomedical staff without balancing it with the perceptions of other key players such as alternative providers. Accounts from hospital staff will be coloured by values and concerns of the cultural system of biomedicine. This may have led, for instance, to overly critical accounts of alternative providers. Thus, further research is required which examines the viewpoints and actual practices of all providers involved. Husbands’ and in-laws’ perspectives on CS and their role in CS decisions merit further attention too before we can make firm programmatic and policy recommendations. More so, the tension between aversion and over-use needs to be explored in more depth since it raises additional concerns about the extent to which CSs are conducted with informed consent. Studies should be conducted into the practice of advising women to undergo CS, how decisions are reached and informed consent is obtained, and women's perceptions and experiences of the procedure.

Nevertheless*,* we can begin to offer some practical recommendations, based in part on our participants’ and other authors’ suggestions. First of all, CS costs should be reduced since they appear to underpin CS refusal, marital instability post CS, and use of alternative providers. This may be particularly persuasive for poorer families who, as reported make less use of *CS*; this policy intervention would redress inequity.

Furthermore*,* as previously reported [52], we found that people may not be fully aware of the reasons why a CS may be advised or medically necessary. Moreover, Vaginal Birth after C-section (VBAC) is practiced in Nigeria as long as a woman’s medical history and clinical condition at the point of labour does not contradict that. It appears however that not everybody is aware of this. More information on what CS is, and what it is not, and that it is not merely for women who are categorically unable to have a vaginal delivery, may redress negative perceptions and enhance acceptance by communities and thus women. This would be one way in which medical providers can address the socio-cultural experience of illness as well as disease [26]. Such health education on CS should target clients recommended for CS and their relatives, including their spouse; antenatal clients; and the wider community. Community based health education should involve a critical conscientization process [53] which fosters critical reflection on social determinants leading to inequalities in health, including women’s restricted gender roles and autonomy. However, changing deep rooted gender ideologies is challenging and will take time*.*

Importantly, considering that alternative providers enjoy communities’ trust and confidence, greater collaboration is essential. Programmes should involve alternative providers so as to guide their activities, and perhaps use their influence to bridge the gap between CS and the prevalent socio-cultural norms that are at odds with CS. For instance, there is a need to explore the scope for integrating spiritual care in the health system [54]. In addition, since it will take time to increase uptake of CS and biomedical maternity services in general, deploying skilled birth attendants in church clinics appears a sensible compromise in the short term.

## Conclusion

Comprehensive, emergency obstetrics care, including CS, is seen as a key factor in the reduction of maternal mortality in low and middle income settings but it is essential that services are available, affordable and accessible. Like previous studies, our findings suggest that, even when available and in principle affordable, gendered socio-cultural obstacles may hinder or delay women from accessing cEmOC including CS, especially in a system which includes powerful alternative providers who offer alternatives. It is only by recognizing and addressing some of these socio-cultural, gendered barriers that appropriate, timely and effective use of CS services can be facilitated and that greater progress towards maternal health goals can be made.

## Footnotes

1 We acknowledge however that the boundaries between Christianity, Islam and African tradition are porous and that Christians and Muslims too are likely to be ‘traditionalist’ to some extent as indeed our analysis demonstrates.

2 an adapted form of English spoken by both the educated and the non-educated across the country and used by majority of Nigerians to conduct day-to-day transactions

3 meaning her husband’s other wife
